# Development and external validation of a machine learning model for predicting 28-day mortality in patients with acute myocardial infarction complicated by malignant arrhythmia: a study using the MIMIC database and a Chinese cohort

**DOI:** 10.3389/fcvm.2026.1908251

**Published:** 2026-07-29

**Authors:** Jiechun Yao, Chun Lin, Qingbo Xu, Guode Li, Jinmei Pan, Jiancheng Wang, Yan Lin

**Affiliations:** 1 Cardiac and Circulatory Medicine Section, Maoming People’s Hospital, Maoming, Guangdong, China; 2 Internal Medicine Division, Maoming People’s Hospital, Maoming, Guangdong, China; 3 Information Technology Department, Maoming People’s Hospital, Maoming, Guangdong, China; 4 Department of Cardiovascular Medicine, Affiliated Hospital of Guangdong Medical University, Zhanjiang, Guangdong, China

**Keywords:** 28-day mortality, acute myocardial infarction, machine learning, malignant ventricular arrhythmia, risk prediction model

## Abstract

**Background:**

Acute myocardial infarction (AMI) complicated by malignant ventricular arrhythmia (MVA) carries high 28-day mortality. Existing risk scores inadequately capture this population. We aimed to develop and externally validate an interpretable machine learning model for predicting 28-day mortality in AMI-MVA patients.

**Methods:**

This retrospective study included 952 AMI-MVA patients from MIMIC-IV (training *n* = 668, internal validation *n* = 284) and 100 patients from Maoming People's Hospital, China (external validation). Feature selection integrated multivariable logistic regression, LASSO, and Boruta, yielding eight predictors: age, lactate, fasting blood glucose, RDW, antiplatelet agents, beta-blockers, ACEI/ARB, and norepinephrine. Seven machine learning algorithms were compared by AUC, calibration, and decision curve analysis. Treatment-free sensitivity models, propensity-score matching (PSM), and inverse-probability-of-treatment-weighting (IPTW) were performed to assess confounding by indication.

**Results:**

28-day mortality was 32.46% (309/952). The SVM model (linear kernel, *C* = 0.1) achieved AUC 0.865 (95% CI 0.824–0.909) in internal validation and 0.898 (95% CI 0.835–0.949) externally, with excellent calibration (Brier score 0.128). SHAP analysis identified beta-blockers, lactate, and ACEI/ARB as the three most influential predictors. A treatment-free sensitivity model (biomarkers only) achieved AUC 0.801 (95% CI 0.748–0.851) internally and 0.771 (95% CI 0.675–0.853) externally. PSM and IPTW attenuated but did not eliminate treatment-outcome associations, consistent with a mixture of genuine prognostic signal and residual confounding.

**Conclusion:**

This interpretable SVM model supports bedside risk stratification for AMI-MVA patients within the first 24 h of ICU admission. The model should be understood as a prognostic snapshot rather than an early prediction tool. Prospective, multicentre validation with standardised severity scores and coronary-anatomy variables is required before routine clinical use.

## Introduction

Acute myocardial infarction (AMI) complicated by malignant ventricular arrhythmia (MVA)—including sustained ventricular tachycardia (VT) and ventricular fibrillation (VF)—represents one of the most immediately life-threatening scenarios in contemporary critical care cardiology (1, 2). 28-day mortality in this population remains high, commonly reaching 26%–38%, and is substantially higher than that observed in AMI patients without malignant arrhythmic complications; patients who develop electrical storm have an even poorer prognosis (2). During the narrow and clinically decisive window of the first 24 h, early identification of patients at extreme mortality risk is essential for timely escalation of monitoring intensity, hemodynamic support, mechanical circulatory support, and individualized antiarrhythmic management (3). However, currently available risk stratification tools are not well tailored to this population. The GRACE and TIMI scores were originally developed for the broader acute coronary syndrome (ACS) population and show limited discriminative performance in the electrophysiologically and metabolically unstable AMI-MVA subgroup, with reported AUC values of approximately 0.72–0.74 (4, 5). Moreover, these scores may exhibit calibration drift across heterogeneous populations and healthcare systems (6). Therefore, there remains an urgent need for a purpose-built and rigorously validated risk stratification tool specific to AMI-MVA patients.

Cardiovascular disease remains the leading cause of global mortality, with ischemic heart disease serving as the primary contributor (7).In China, this burden is particularly substantial, with more than 3 million AMI events estimated annually; despite advances in reperfusion strategies and intensive cardiovascular care, mortality among critically ill AMI subsets remains elevated (8). The pathophysiology of MVA complicating AMI is time-dependent and heterogeneous. Early-onset ventricular arrhythmias are predominantly driven by acute ischemia-reperfusion injury, oxidative stress, calcium overload, and autonomic instability, whereas late-onset events are more closely related to myocardial scar formation, electrical remodelling, and persistent arrhythmogenic substrate (9). Importantly, this arrhythmic vulnerability is closely intertwined with systemic metabolic derangement. Stress hyperglycemia, often quantified by the stress hyperglycemia ratio (SHR), and hyperlactatemia reflect autonomic stress, impaired substrate utilization, and end-organ oxygen debt, and have been associated with both MVA occurrence and adverse in-hospital outcomes (10, 11). Similarly, red cell distribution width (RDW), an accessible marker integrating systemic inflammation, oxidative stress, and hematopoietic dysfunction, has been consistently linked to poor prognosis after AMI (12). These observations suggest that AMI-MVA should not be regarded solely as an electrical event, but rather as a multidimensional critical syndrome arising from the convergence of electrophysiological instability, metabolic dysregulation, systemic inflammation, and tissue hypoperfusion.

Recognition of these complex interactions has encouraged the development of more disease-specific prediction models. For example, D'Ascenzo et al. (13) developed the PRAISE model, a machine learning-based framework incorporating clinical and laboratory variables to predict adverse outcomes after ACS. Sun et al. (14)constructed a logistic regression-based model to predict ventricular arrhythmia risk in AMI patients. Nevertheless, existing studies still leave several important methodological and clinical gaps. First, metabolic and systemic stress dimensions remain underrepresented in AMI-MVA prediction research; clinically available markers such as lactate, fasting blood glucose, and RDW are seldom integrated within a unified mortality prediction framework. Second, external validation remains insufficient. As emphasized by Wynants et al. (15) and Steyerberg et al. (16), prediction models derived from single-centre data without independent validation, adequate calibration assessment, and decision-curve evaluation are vulnerable to overfitting and often have limited generalisability across institutions. Third, conventional regression-based models may not fully capture nonlinear relationships and high-order interactions among metabolic markers, treatment status, and hemodynamic instability. Contemporary machine learning algorithms—including random forests, XGBoost, support vector machines (SVM), and artificial neural networks—offer methodological advantages in modelling such complex associations, and previous work has shown that gradient boosting models can outperform GRACE scores in predicting post-AMI mortality (17). However, algorithmic complexity alone is insufficient; as Christodoulou et al. (18) cautioned, predictive gains from machine learning must be balanced against methodological rigor, transparency, and clinical interpretability. Although Cao et al. (19) recently explored mortality prediction in AMI complicated by ventricular arrhythmia, their study was limited by a small sample size, single-centre design, and reliance on traditional linear modelling. Therefore, a prediction strategy integrating advanced machine learning, interpretable artificial intelligence, and robust external validation remains needed for AMI-MVA patients.

The MIMIC-IV database provides a suitable platform for addressing these gaps, offering large-scale, heterogeneous, and high-resolution electronic health records from critically ill patients (20). Its standardised structure and open-access framework support reproducible model development and allow the integration of demographic characteristics, laboratory parameters, treatment information, and critical care variables. At the same time, the clinical adoption of machine learning models requires interpretability. SHapley Additive exPlanations (SHAP) can quantify both global feature importance and individualized feature contributions, thereby linking model outputs with clinically meaningful reasoning and reducing the perceived “black box” barrier (21).

To our knowledge, the current literature lacks a comprehensive framework for AMI-MVA patients that simultaneously incorporates multidimensional metabolic and treatment-related predictors, compares multiple machine learning algorithms with interpretable outputs, and validates model performance in an independent cohort from a geographically and ethnically distinct healthcare setting. To address this unmet need, the present study developed machine learning-based models for predicting 28-day mortality among AMI-MVA patients using the MIMIC-IV database as the development cohort, and externally validated the optimal model in an independent Chinese cohort. Model performance was comprehensively assessed in terms of discrimination, calibration, and clinical net benefit, in accordance with contemporary TRIPOD + AI reporting principles (22, 23). This framework aims to provide a robust, interpretable, and externally validated tool to support early bedside risk stratification within the first 24 h of ICU admission in AMI-MVA patients across diverse clinical settings.

The model targets patients with documented MVA within the first 24 h of ICU admission for acute myocardial infarction, supporting bedside triage and short-term prognostic assessment. It is not designed to predict MVA onset in the broader AMI population.

## Methods

### Data source

Patient records for this retrospective analysis were drawn from version 3.1 of the MIMIC-IV (Medical Information Mart for Intensive Care IV) database. This resource compiles de-identified records from admissions to Beth Israel Deaconess Medical Center spanning 2008 to 2022. As all identifiers had been stripped, the need for informed consent did not apply. Database queries were carried out by Jiechun Yao, who held credentialed access after completing CITI training (Certificate ID: 72482547). The external validation sample comprised AMI-MVA patients treated at Maoming People's Hospital, Guangdong, China, with records spanning January 2021 through December 2024. The institutional review board of Maoming People's Hospital approved the external study before any data were collected (Approval ID: PJ2026MI-K049-02). Because the design was retrospective and relied on fully anonymised records that prevent patient identification, informed consent was again waived. Patient confidentiality was safeguarded throughout, and every record was rigorously de-identified.

### Study population

Cases of AMI with concurrent malignant arrhythmia were retrieved through ICD-9/10 diagnostic coding (24). For AMI, qualifying ICD-9 codes were 41,000–41,092, and ICD-10 codes were I21, I210-I214, I219, I21A, I21A1, and I21A9. MVA encompassed sustained ventricular tachycardia (episodes exceeding 30 s), ventricular flutter, and ventricular fibrillation, captured by ICD-9 codes 4,271, 42,741, and 42,742 or ICD-10 codes I472, I490, I4901, and I4902(2).

Eligibility for the MIMIC-IV cohort required: (1) an age of at least 18 years, (2) a first ICU stay, and (3) a coded diagnosis of AMI with malignant arrhythmia under the codes listed above. Patients were excluded when they (1) were younger than 18 years, (2) had a prior ICU admission, (3) lacked a confirmed AMI or malignant arrhythmia diagnosis, or (4) had incomplete data for key variables. This process yielded 952 eligible patients from MIMIC-IV, with the full screening workflow shown in Supplementary Figure S1.

Using Structured Query Language (SQL) queries for the MIMIC-IV database, we extracted demographic information including age, gender, height, weight, and body mass index (BMI); comorbidities including hypertension, diabetes mellitus, congestive heart failure (CHF), chronic kidney disease (CKD), chronic obstructive pulmonary disease (COPD), sepsis, acute kidney injury (AKI), smoking, and alcohol abuse; severity scores including Acute Physiology Score III (APS III), Sequential Organ Failure Assessment (SOFA), and Glasgow Coma Scale (GCS); vital signs including heart rate, respiratory rate, systolic blood pressure (SBP), diastolic blood pressure (DBP), peripheral oxygen saturation (SpO₂), and temperature; laboratory parameters including pH, base excess (BE), lactate, white blood cell count (WBC), hematocrit, hemoglobin, RDW, platelets, creatinine, blood urea nitrogen (BUN), glucose, fasting blood glucose (FBG)（FBG was operationally defined as the first fasting glucose measurement obtained after hospital admission.）, anion gap, sodium, potassium, chloride, bicarbonate, total calcium, magnesium, phosphate, prothrombin time (PT), activated partial thromboplastin time (APTT), international normalized ratio (INR), cardiac troponin T (cTnT), creatine kinase-myocardial band (CK-MB), alanine aminotransferase (ALT), aspartate aminotransferase (AST), and alkaline phosphatase (ALP); and treatment records including aspirin, beta-blockers, statins, angiotensin-converting enzyme inhibitors/angiotensin receptor blockers (ACEI/ARB), norepinephrine, dopamine, milrinone, percutaneous coronary intervention (PCI), cardiopulmonary resuscitation (CPR), invasive mechanical ventilation (IMV), and continuous renal replacement therapy (CRRT). For variables with multiple measurements, such as vital signs or laboratory results, within 24 h of ICU admission, we extracted the first available value. This data extraction strategy was consistently applied to the external validation cohort from Maoming People's Hospital. Medication-exposure variables (antiplatelet, beta-blocker, ACEI/ARB, norepinephrine) were coded as documented administration during the first 24 h of ICU admission. They therefore represent a binary summary of early exposure and do not capture initiation timing, dose adjustment, or discontinuation. This choice preserves interpretability and cross-cohort harmonization; a time-varying analysis was not feasible given the granularity of available medication records. To preserve comparability, the external Maoming cohort applied identical eligibility rules and ultimately contributed 100 patients. The primary endpoint was 28-day mortality after ICU admission, defined as death from any cause within 28 days of the index ICU stay. Because the external cohort was assembled solely for validation, only the eight final predictors and the outcome were extracted, in keeping with the streamlined validation strategy advocated by Steyerberg et al (6).

#### Cohort assembly ICD codes

MVA events were identified using the following ICD-9/10 codes: sustained ventricular tachycardia (ICD-9 427.1/ICD-10 I47.2), ventricular fibrillation and flutter (ICD-9 427.41, 427.42/ICD-10 I49.01, I49.02). The full code list and clinical meanings are given in Supplementary Table S7. External cohort admission unit. At Maoming People's Hospital, patients with confirmed AMI complicated by MVA were admitted to the coronary care unit (CCU) or the medical intensive care unit (MICU) based on the presenting predominant organ failure and bed availability. Of the 100 external-cohort patients, 76 (76%) were admitted directly to the CCU and 24 (24%) to the MICU.

#### Imputation

The proportion of missing values for each variable is reported in Supplementary Table S1. Variables with a missing rate below 50% were retained and imputed using multiple imputation by chained equations (MICE, 20 imputations, predictive-mean matching); variables exceeding 50% missingness were excluded. Lactate had a missingness rate of 44.4% in the MIMIC-IV cohort — below the threshold but relatively high. Given its established role as a core marker of tissue hypoperfusion and its consistently reported strong prognostic value in AMI-MVA populations, lactate was retained. To assess imputation robustness, we performed a complete-case sensitivity analysis restricted to patients with actually measured lactate; the complete-case subset (*n* = 532, 28-day mortality 38.5%) yielded an AUC of 0.879 vs. 0.851 in the MICE-imputed main model (*n* = 952, mortality 32.5%), a difference of 0.028 (bootstrap 95% CI −0.09 to 0.05). The narrow overlap and non-significant difference support the reasonableness of the MICE-imputed values, while the higher mortality in the complete-case subset itself suggests informative missingness (see Limitations). In the main analysis, imputation was fitted on the full MIMIC-IV cohort before the 80/20 training/validation split; this is a conservative pragmatic choice for a small-cohort ICU database but risks optimistic bias because information from the validation set can influence the training-set imputation. To quantify this bias, we performed a training-set-only sensitivity analysis in which MICE was fitted on the 80% training set alone and the locked imputation model was applied to the internal validation set and to the external cohort (Supplementary Section S13). We report both estimates transparently and recommend the more conservative training-only estimate where a single value is required.

Adequacy of the training-set sample size was judged against the established rule of ten events per variable (EPV). With eight predictors retained, the model demanded a minimum of 80 outcome events. Given the observed mortality rate, roughly 217 28-day deaths fell within the training set, producing an EPV near 27.1, comfortably above the threshold and consistent with stable estimation.

### Statistical analysis

Owing to skewed distributions, continuous measures were reported as medians with interquartile ranges and contrasted via the Wilcoxon rank-sum test. Categorical measures appeared as counts with percentages and were tested using the chi-square or Fisher's exact test. Statistical significance was set at a two-sided *P* below 0.05.

The MIMIC-IV cases were partitioned at random into training (*n* = 668) and internal validation (*n* = 284) subsets, following a 7:3 allocation stratified by outcome.

Predictor identification was confined to the training set and followed a three-tier strategy designed for reproducibility. The first tier used univariate testing to flag variables differing between survivors and non-survivors (*P* < 0.05). The second tier carried the significant variables forward into multivariable logistic regression to isolate independent predictors. The third tier ran two methods in parallel: the Boruta algorithm, a wrapper approach that gauges importance through Z-scores against randomised shadow features, and LASSO regression, whose tuning parameter (*λ*) was set by cross-validation. Only variables shared by all three procedures, their intersection, entered the final predictor set (excluded candidate variables are listed in Supplementary Table S11), ensuring agreement across independent analyses.

### Model development and validation

SHAP analysis was used to render the best-performing model interpretable. Bar and beeswarm displays conveyed global feature importance and showed how strongly, and in which direction, each variable shaped predictions, thereby improving transparency for clinical readers. Finer detail came from SHAP dependence plots together with patient-level waterfall and force plots. Transferability was then examined in the independent Maoming People's Hospital cohort, which probed performance under a different population and data environment. SHAP values express predictive association, not causal effect; causal interpretation would require a target-trial design beyond the scope of this study.

### Model interpretability and external validation

For the SVM model, the grid search explored regularization parameter C ∈ {0.1, 1}, kernel ∈ {linear, RBF}, gamma ∈ {scale, auto, 0.01, 0.1}, and polynomial degree ∈ {2, 3}. The optimal configuration was: kernel = linear, C = 0.1, gamma = scale, degree = 2.

Within the internal validation set, performance was characterised through ROC curves, calibration plots, and decision curve analysis (DCA). We computed the area under the ROC curve (AUC) together with accuracy, precision, sensitivity, specificity, and F1-score to gauge predictive capacity. Model choice rested on the balance of these metrics, particularly stable discrimination, sound calibration, and clinical utility. On this basis, the SVM model delivered the steadiest overall behaviour and was adopted as the final model.

The selected SVM model underwent external testing in the independent Maoming People's Hospital cohort. The same panel of metrics (AUC, accuracy, precision, sensitivity, specificity, F1-score), supplemented by calibration curves and DCA, confirmed its generalisability and clinical relevance. The tool was therefore conceived as an early bedside 28-day mortality risk estimator updated during admission, rather than a fixed admission-only prognostic score.

### Reporting guidelines

This manuscript follows established reporting principles for clinical prediction models. The study was informed by the TRIPOD-AI recommendations for transparent reporting of AI-based prediction models and the PROBAST-AI framework for bias and applicability assessment. Given the retrospective observational design, CONSORT-AI was not applicable.

All analyses were conducted using R version 4.5.1 and Python version 3.10.4.

## Results

### Baseline characteristics

From MIMIC-IV, 952 AMI-MVA patients satisfied the inclusion criteria and were allocated at random to a training cohort (*n* = 668) and an internal validation cohort (*n* = 284). Across the whole sample, 309 patients (32.46%) died within 28 days. Table 1 Table 1 summarises baseline characteristics, with training-set values given in Supplementary Table S5. Relative to survivors, non-survivors tended to be older and carried markedly higher severity scores (APS III and SOFA). They also showed pronounced metabolic and haematological derangements, conspicuously raised lactate, FBG, and RDW, alongside signs of severe organ injury such as elevated creatinine and cTnT and a lower pH. Critical comorbidities, notably sepsis and AKI, were more frequent among non-survivors, as was the need for vasoactive support, particularly norepinephrine. By contrast, these patients underwent PCI less often and received standard cardioprotective drugs (antiplatelet agents, beta-blockers, and ACEI/ARB) far less frequently. In the external Maoming People's Hospital cohort of 100 patients, 53 (53%) reached the primary endpoint of 28-day mortality. Supplementary Table S6 reports the external cohort's baseline profile by survival status.

**Table 1:** Baseline characteristics of the study population.

Characteristics	Overall (*n* = 952)	Survivors (*n* = 643)	Non-survivors (*n* = 309)	*P* value
Demographics				
Age, years	70.0 [60.8, 78.0]	68.0 [59.0, 77.0]	74.0 [63.0, 82.0]	<0.001
Gender, male, *n* (%)	708 (74.4)	491 (76.4)	217 (70.2)	0.051
Height, cm	173.0 [164.5, 178.0]	173.0 [165.0, 180.0]	170.0 [163.0, 178.0]	0.019
Weight, kg	83.0 [71.1, 98.0]	84.0 [72.0, 98.2]	81.0 [69.1, 96.5]	0.105
BMI, kg/m^2^	28.3 [25.3, 31.9]	28.3 [25.5, 31.8]	27.8 [24.7, 32.4]	0.337
Comorbidities, *n* (%)				
Hypertension	297 (31.2)	214 (33.3)	83 (26.9)	0.054
Diabetes mellitus	384 (40.3)	235 (36.6)	149 (48.2)	0.001
CHF	632 (66.4)	422 (65.6)	210 (68.0)	0.522
CKD	316 (33.2)	191 (29.7)	125 (40.5)	0.001
COPD	223 (23.4)	144 (22.4)	79 (25.6)	0.317
Sepsis	509 (53.5)	306 (47.6)	203 (65.7)	<0.001
AKI	787 (82.7)	507 (78.9)	280 (90.6)	<0.001
Smoking	49 (5.2)	33 (5.1)	16 (5.2)	1
Alcohol abuse	3 (0.3)	2 (0.3)	1 (0.3)	1
Severity scores				
APS III score	47.0 [34.0, 63.0]	40.0 [30.0, 54.0]	62.0 [49.0, 79.0]	<0.001
SOFA score	6.0 [3.0, 10.0]	5.0 [2.0, 9.0]	9.0 [6.0, 12.0]	<0.001
GCS	14.0 [10.0, 15.0]	14.0 [13.0, 15.0]	14.0 [7.0, 15.0]	<0.001
Vital signs				
Heart rate, beats/min	86.0 [72.8, 99.0]	85.0 [72.0, 98.0]	88.0 [74.0, 101.0]	0.061
Respiratory rate, breaths/min	19.0 [16.0, 23.2]	18.0 [16.0, 22.0]	20.0 [18.0, 25.0]	<0.001
SBP, mmHg	116.0 [101.0, 131.0]	118.0 [103.0, 132.0]	111.0 [96.0, 127.0]	<0.001
DBP, mmHg	68.0 [57.0, 80.0]	70.0 [59.0, 81.0]	66.0 [53.0, 76.0]	<0.001
SpO2, %	98.0 [95.0, 100.0]	98.0 [95.0, 100.0]	98.0 [95.0, 100.0]	0.509
Temperature,°C	36.6 [36.3, 36.9]	36.6 [36.4, 36.9]	36.5 [36.1, 36.8]	<0.001
Laboratory findings				
pH	7.36 [7.29, 7.41]	7.37 [7.31, 7.41]	7.33 [7.24, 7.40]	<0.001
BE, mmol/L	−2.0 [−6.0, 0.0]	−1.0 [−5.0, 0.0]	−5.0 [−9.0, −1.0]	<0.001
Lactate, mmol/L	1.8 [1.2, 3.2]	1.7 [1.2, 2.3]	2.9 [1.6, 6.2]	<0.001
WBC, 10^9^/L	12.6 [9.3, 17.4]	11.9 [9.1, 16.4]	14.2 [10.0, 19.4]	<0.001
Hematocrit, %	35.2 [29.5, 39.6]	35.9 [29.8, 40.5]	33.2 [28.8, 38.5]	0.001
Hemoglobin, g/dL	11.6 [9.7, 13.3]	11.9 [10.1, 13.5]	10.9 [9.2, 12.7]	<0.001
RDW, %	14.2 [13.3, 15.8]	14.0 [13.2, 15.2]	14.9 [13.8, 16.7]	<0.001
Platelets, 10^9^/L	212.5 [162.0, 270.0]	214.0 [164.0, 265.0]	205.0 [153.0, 277.0]	0.584
Creatinine, mg/dL	1.2 [0.9, 1.9]	1.1 [0.9, 1.6]	1.6 [1.2, 2.9]	<0.001
BUN, mg/dL	23.5 [16.0, 41.0]	21.0 [15.0, 33.0]	34.0 [21.0, 56.0]	<0.001
Glucose, mg/dL	154.0 [120.0, 219.0]	144.0 [116.0, 199.5]	178.0 [135.0, 258.0]	<0.001
FBG, mg/dL	139.0 [113.0, 188.0]	131.0 [112.0, 168.5]	163.0 [125.0, 216.0]	<0.001
Anion gap, mmol/L	15.0 [13.0, 19.0]	15.0 [12.0, 17.0]	18.0 [14.0, 22.0]	<0.001
Sodium, mmol/L	138.0 [135.0, 140.0]	138.0 [135.0, 140.0]	138.0 [134.0, 141.0]	0.985
Potassium, mmol/L	4.2 [3.8, 4.7]	4.2 [3.8, 4.6]	4.4 [3.9, 5.0]	<0.001
Chloride, mmol/L	103.0 [99.0, 106.0]	103.0 [99.0, 106.0]	102.0 [97.0, 107.0]	0.085
Bicarbonate, mmol/L	22.0 [19.0, 24.0]	22.0 [20.0, 25.0]	20.0 [17.0, 23.0]	<0.001
Total calcium, mg/dL	8.5 [8.0, 9.0]	8.5 [8.0, 9.0]	8.4 [7.8, 8.9]	0.08
Magnesium, mg/dL	2.1 [1.9, 2.3]	2.0 [1.8, 2.2]	2.2 [1.9, 2.4]	<0.001
Phosphate, mg/dL	3.8 [3.1, 4.9]	3.6 [2.9, 4.4]	4.4 [3.7, 6.1]	<0.001
PT, (s)	14.2 [12.6, 17.5]	13.6 [12.3, 16.4]	15.6 [13.4, 22.5]	<0.001
APTT, (s)	40.4 [29.6, 71.5]	37.8 [29.2, 64.8]	47.3 [31.2, 86.2]	<0.001
INR	1.3 [1.1, 1.6]	1.2 [1.1, 1.5]	1.4 [1.2, 2.1]	<0.001
cTnT, ng/mL	0.90 [0.26, 3.14]	0.81 [0.22, 2.80]	1.14 [0.33, 3.73]	0.01
CK-MB, U/L	24.0 [7.0, 107.5]	25.0 [7.0, 113.0]	23.0 [7.0, 79.0]	0.283
ALT, U/L	58.0 [28.0, 151.0]	52.0 [25.0, 117.0]	80.0 [35.0, 202.0]	<0.001
AST, U/L	114.5 [49.0, 302.2]	106.0 [46.0, 263.0]	139.0 [54.0, 396.0]	0.001
ALP, U/L	81.0 [62.0, 113.2]	78.0 [60.0, 99.0]	90.0 [67.0, 138.0]	<0.001
Treatments, *n* (%)				
Antiplatelet agents	813 (85.4)	581 (90.4)	232 (75.1)	<0.001
Beta-blockers	718 (75.4)	568 (88.3)	150 (48.5)	<0.001
Statins	157 (16.5)	110 (17.1)	47 (15.2)	0.519
ACEI/ARB	388 (40.8)	350 (54.4)	38 (12.3)	<0.001
Norepinephrine	513 (53.9)	280 (43.6)	233 (75.4)	<0.001
Dopamine	224 (23.5)	110 (17.1)	114 (36.9)	<0.001
Milrinone	96 (10.1)	71 (11.0)	25 (8.1)	0.193
PCI	147 (15.4)	118 (18.4)	29 (9.4)	<0.001
CPR	65 (6.8)	36 (5.6)	29 (9.4)	0.042
IMV	519 (54.5)	302 (47.0)	217 (70.2)	<0.001
CRRT	109 (11.4)	38 (5.9)	71 (23.0)	<0.001

Continuous variables are presented as median [interquartile range (IQR)], and categorical variables are presented as numbers (percentages). ACEI, angiotensin-converting enzyme inhibitor; AKI, acute kidney injury; ALP, alkaline phosphatase; ALT, alanine aminotransferase; APS III, Acute Physiology Score III; APTT, activated partial thromboplastin time; ARB, angiotensin II receptor blocker; AST, aspartate aminotransferase; BE, base excess; BMI, body mass index; BUN, blood urea nitrogen; CHF, congestive heart failure; CK-MB, creatine kinase-myocardial band; CKD, chronic kidney disease; COPD, chronic obstructive pulmonary disease; CPR, cardiopulmonary resuscitation; CRRT, continuous renal replacement therapy; cTnT, cardiac troponin T; DBP, diastolic blood pressure; FBG, fasting blood glucose; GCS, Glasgow Coma Scale; IMV, invasive mechanical ventilation; INR, international normalized ratio; IQR, interquartile range; PCI, percutaneous coronary intervention; PT, prothrombin time; RDW, red blood cell distribution width; SBP, systolic blood pressure; SOFA, Sequential Organ Failure Assessment; SpO2, peripheral capillary oxygen saturation; WBC, white blood cell.

In a stratified analysis of the derivation cohort by MVA subtype, 887 (93.2%) patients had sustained VT only and 65 (6.8%) had VF or pulseless VT (Supplementary Section S8, identified by CPR documentation as a proxy). 28-day mortality was 31.6% in the VT-only subgroup and 44.6% in the VF/pulseless-VT subgroup. Subtype-stratified model discrimination is presented in Supplementary Table S9 (AUC VT-only 0.859; VF/pulseless-VT 0.948, noting the very small subgroup size of *n* = 18 in validation).

### Feature selection

**Figure 1:**
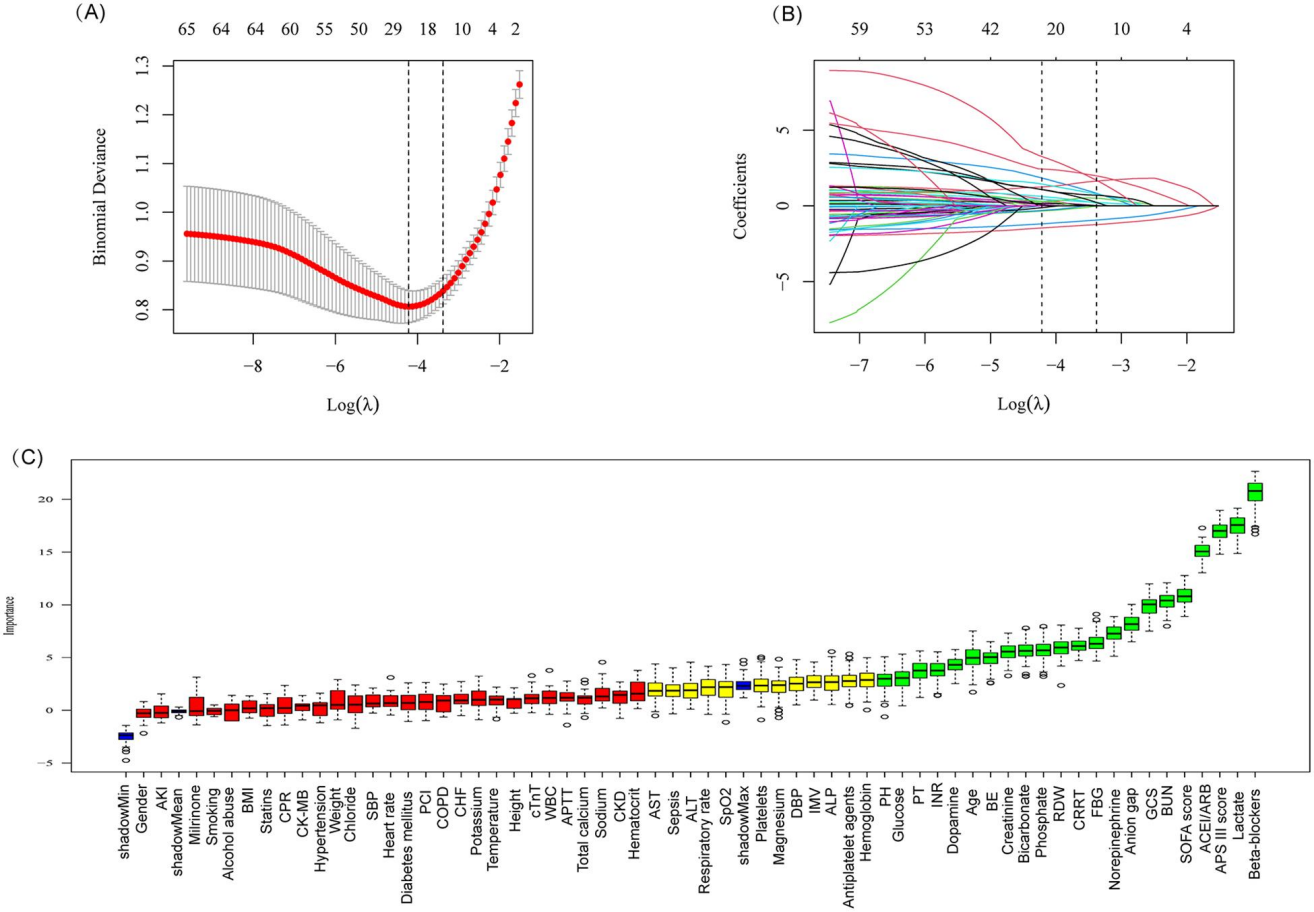
Feature selection using LASSO regression and the boruta algorithm **(A)** Ten-fold cross-validation curve for tuning the penalty parameter (*λ*) in the LASSO model. The *x*-axis represents log (*λ*), and the *y*-axis shows the binomial deviance (error). The solid red line indicates the cross-validated error curve, with the grey error bars representing the 95% confidence intervals. The two vertical dashed lines indicate the *λ*_min_ and *λ*_1se_ values. At *λ*_1se_ = 0.03409493, the model is optimally regularized, identifying a total of 15 clinically relevant variables with non-zero coefficients (Supplementary Table S4). **(B)** Coefficient shrinkage paths for the LASSO model under varying penalty strengths. The *x*-axis is log(*λ*), and the *y*-axis shows the coefficient profiles. Each curve represents a distinct clinical feature. As the penalty increases (moving from left to right), the coefficients shrink toward zero, retaining only the most influential predictors. **(C)** Results of the Boruta feature-importance analysis. Boxplots compare the importance distribution (*y*-axis) of each clinical variable with its corresponding shadow features (blue boxes) generated via random forest iterations. The green boxes represent the 29 “Confirmed” variables whose importance consistently exceeded the maximum shadow importance (Supplementary Table S3). The red and yellow boxes denote “Rejected” and “Tentative” variables, respectively.

Within the training cohort, univariate testing first isolated variables that differed significantly (*P* < 0.05) between survivors and non-survivors. These candidates then entered multivariable logistic regression, which retained 10 independent predictors (Supplementary Table S2). Running in parallel, the Boruta algorithm ranked features by Z-value and confirmed 29 candidate predictors (Figure 1C). LASSO regression was applied concurrently; with the tuning parameter (*λ*) fixed by cross-validation, 15 variables retained non-zero coefficients (Figures 1A,B). We took the intersection of these three independent selectors, multivariable logistic regression, LASSO, and Boruta, as the definitive feature set, a step intended to bolster robustness and generalisability. This converged on 8 core clinical variables, antiplatelet agents, norepinephrine, RDW, lactate, FBG, age, beta-blockers, and ACEI/ARB, which served as the final inputs for the machine learning models (Figure 1).

### Performance comparison of machine learning models

**Figure 2:**
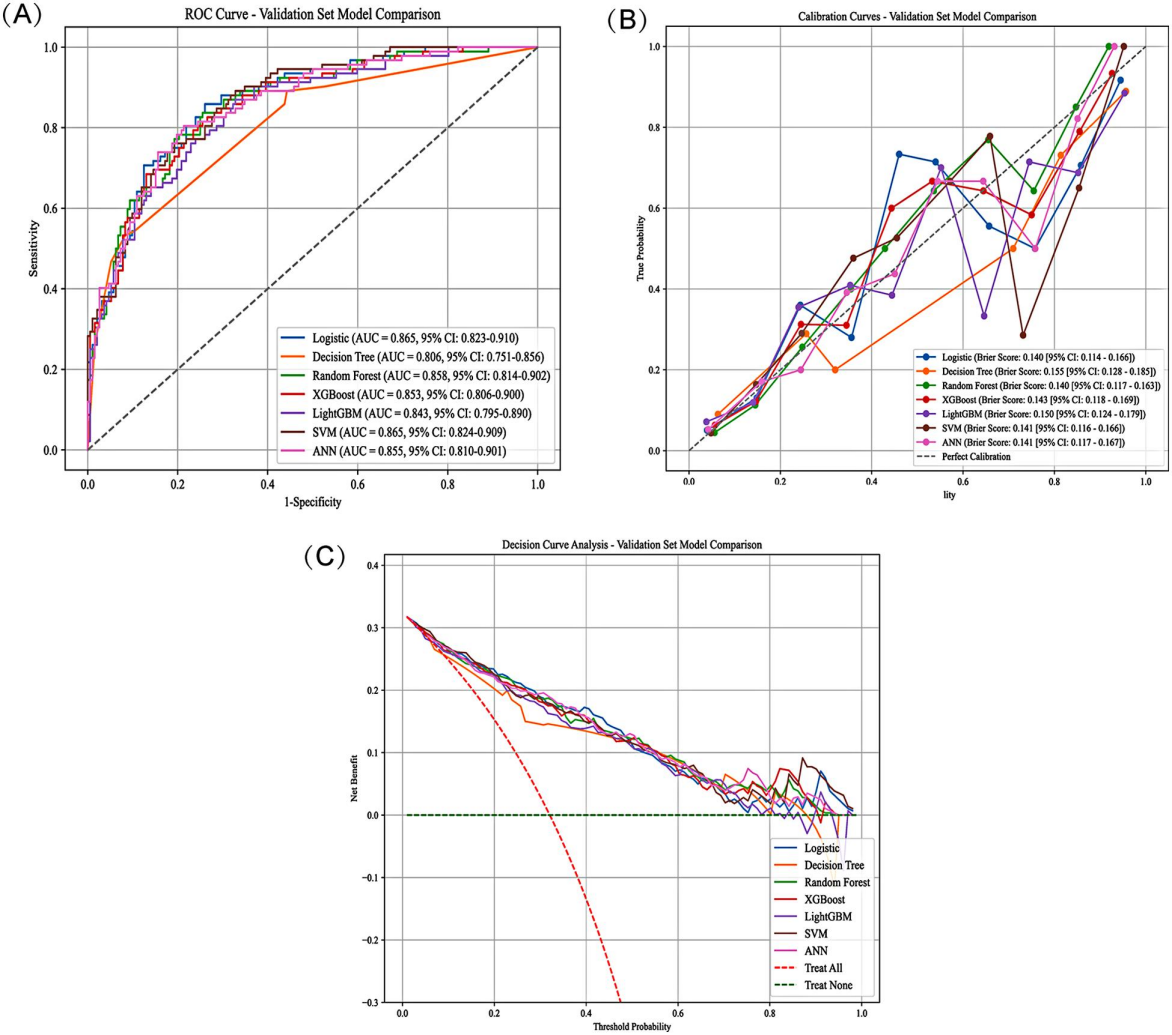
Performance of the machine learning-based modelling frameworks in the validation set. **(A)** Receiver operating characteristic (ROC) curves showing the discriminative ability of the seven machine learning models. **(B)** Calibration curves comparing the model-predicted probabilities (*x*-axis) to the true observed outcomes (*y*-axis). Brier scores with 95% CIs are provided in the legend; lower scores indicate better calibration. **(C)** Decision curve analysis (DCA) illustrating the net clinical benefit (*y*-axis) of the machine learning models across different threshold probabilities (*x*-axis).

Seven algorithms were trained to predict 28-day mortality: logistic regression, decision tree, random forest, XGBoost, LightGBM, SVM, and ANN. Table 2 and Figure 2A compile their metrics. SVM gave the strongest and most balanced discrimination, leading on AUC (0.865; 95% CI 0.824-0.909), accuracy (0.792), and precision (0.654). Sensitivity (0.761) and specificity (0.807) stayed in close balance. Logistic regression matched its discrimination (AUC = 0.865) and reached higher sensitivity (0.859) but trailed on specificity (0.740). Random forest (AUC = 0.858), ANN (AUC = 0.855), and XGBoost (AUC = 0.853) likewise performed well, whereas LightGBM (AUC = 0.843) and the decision tree (AUC = 0.806) discriminated comparatively poorly.

**Table 2:** Assessment of seven predictive models within the internal validation set.

Model	AUC	95% CI Lower	95% CI Upper	Accuracy	Precision	Sensitivity	Specificity	F1 Score
Logistic	0.865	0.823	0.910	0.778	0.612	0.859	0.740	0.715
Decision Tree	0.806	0.751	0.856	0.792	0.754	0.533	0.917	0.624
Random Forest	0.858	0.814	0.902	0.775	0.611	0.837	0.745	0.706
XGBoost	0.853	0.806	0.900	0.764	0.598	0.826	0.734	0.694
LightGBM	0.843	0.795	0.890	0.736	0.559	0.870	0.672	0.681
SVM	0.865	0.824	0.909	0.792	0.654	0.761	0.807	0.704
ANN	0.855	0.810	0.901	0.792	0.643	0.804	0.786	0.715

The SVM (linear kernel, *C* = 0.1) and logistic regression models produced similar overall discrimination (both AUC 0.865 in internal validation). At the Youden-index threshold, SVM prioritized specificity (sensitivity 0.761, 95% CI 0.676–0.845; specificity 0.807, 95% CI 0.749–0.861) while logistic regression prioritized sensitivity (sensitivity 0.859, 95% CI 0.778–0.929; specificity 0.740, 95% CI 0.675–0.801). SHAP analysis and external validation results for both models are reported in Supplementary Section S12.

Calibration plots (Figure 2B) showed that SVM, logistic regression, and random forest aligned closely with observed outcomes, posting the lowest Brier scores (around 0.140 for logistic regression and random forest). XGBoost and LightGBM calibrated moderately well (Brier scores 0.143 and 0.150), while the decision tree drifted furthest from the ideal line (Brier score = 0.155).

Decision curve analysis (Figure 2C) showed SVM and logistic regression yielding the greatest net benefit across a broad, clinically meaningful span of threshold probabilities, clearly surpassing the treat-all and treat-none alternatives. Remaining models gave intermediate benefit, and the decision tree lagged throughout. Taken together, SVM proved the most dependable across discrimination, calibration, accuracy, and clinical value, and was accordingly chosen as the final model for interpretation and external validation.

### External validation of the optimal SVM model

**Figure 3:**
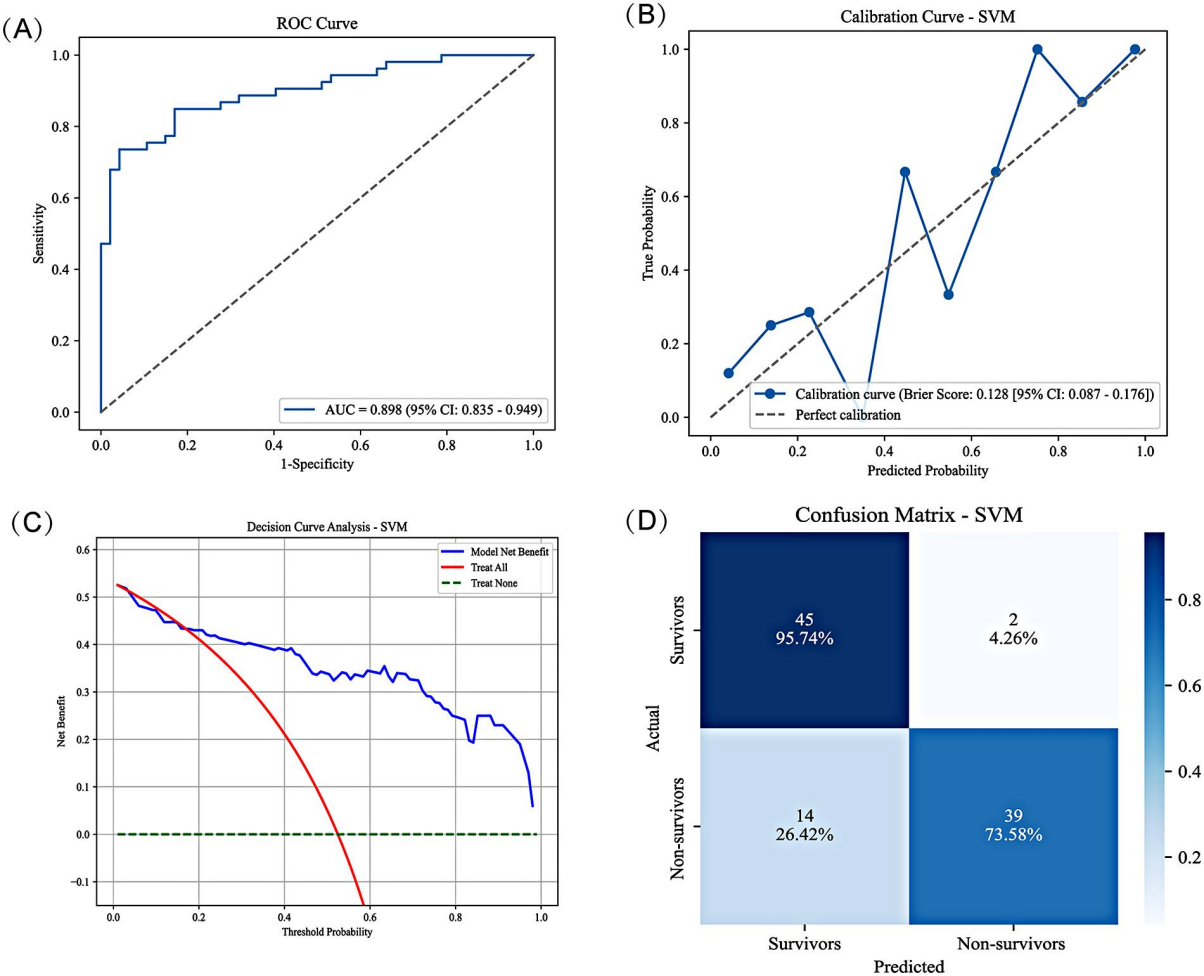
External validation and performance of the machine learning framework. **(A)** ROC curve. **(B)** Calibration curve. **(C)** DCA curve. **(D)** Confusion matrix.

External testing of the chosen SVM model drew on the independent Maoming People's Hospital cohort. Discrimination held firm for 28-day mortality (AUC = 0.898; 95% CI 0.835–0.949; Figure 3A). The model reached an accuracy of 0.840, precision of 0.951, sensitivity of 0.736, specificity of 0.957, and an F1-score of 0.830, identifying both high- and low-risk patients reliably. Calibration was sound (Figure 3B), with predictions tracking observed events (Brier score = 0.128; 95% CI 0.087–0.176). Decision curve analysis (Figure 3C) again favoured the SVM model over treat-all and treat-none strategies across relevant thresholds, pointing to better decision support. The confusion matrix (Figure 3D) confirmed strong overall accuracy: of 100 cases, 39 non-survivors and 45 survivors were classified correctly (TP = 39, TN = 45, FP = 2, FN = 14), consistent with the metrics above.

### SHAP interpretation of the optimal SVM model

**Figure 4:**
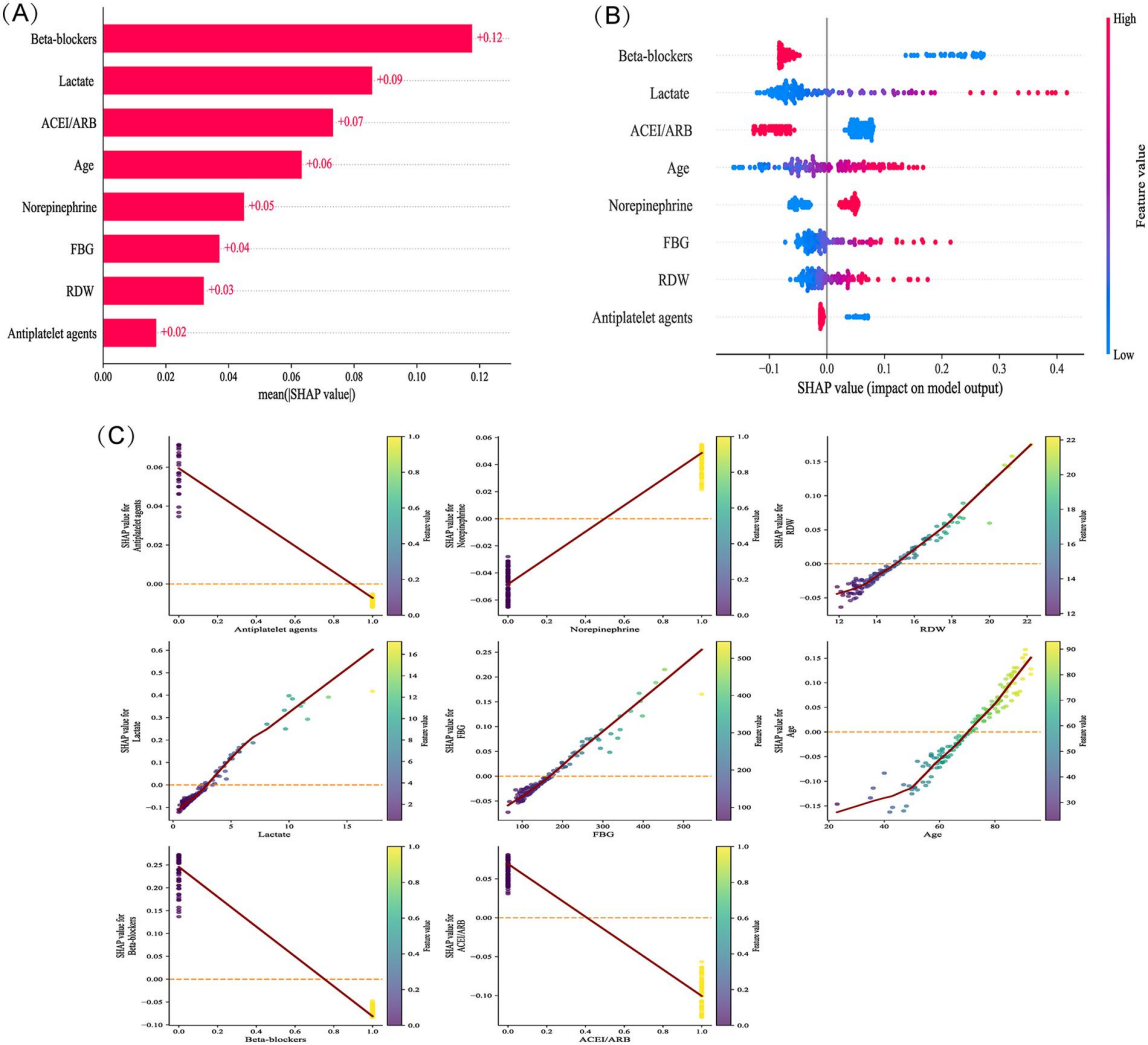
SHAP interpretation of the optimal SVM model on the validation set. **(A)** SHAP Importance Plot showing showing the mean absolute SHAP value for each predictor, ranked by importance. **(B)** SHAP summary (beeswarm) plot. Each dot represents a single patient's SHAP value for a particular feature. The *x*-axis shows the SHAP value, indicating how much a feature's presence or absence changes the model's output prediction for that instance. Red dots indicate high feature values, while blue dots indicate low feature values. **(C)** SHAP dependence (scatter) plots illustrating the relationship between a feature's value and its corresponding SHAP value.

To make the chosen SVM model interpretable and clinically useful, SHAP quantified how each feature shaped its predictions (Figure 4). Ranked by mean absolute SHAP value, the three most influential drivers of 28-day mortality were beta-blockers, lactate, and ACEI/ARB (Figure 4A), with age, norepinephrine, FBG, RDW, and antiplatelet agents also weighing appreciably. The beeswarm summary (Figure 4B) mapped the spread and direction of these contributions. Rising lactate, for example, repeatedly produced positive SHAP values, marking a firm link to higher predicted mortality. Beta-blockers and ACEI/ARB, by contrast, often returned negative SHAP values, in keeping with a protective association, whereas greater age and higher norepinephrine doses pushed predicted risk upward.

SHAP dependence plots dissected how single factors swayed the model's mortality estimates (Figure 4C). Antiplatelet agents and beta-blockers consistently produced negative SHAP values, indicating an association with lower 28-day mortality. Likewise, the absence of norepinephrine corresponded to negative SHAP values and a lower predicted risk. Higher RDW, lactate, FBG, and age moved in the opposite direction, with rising values yielding progressively positive SHAP contributions; the gradient was especially steep for FBG and lactate.

**Figure 5:**
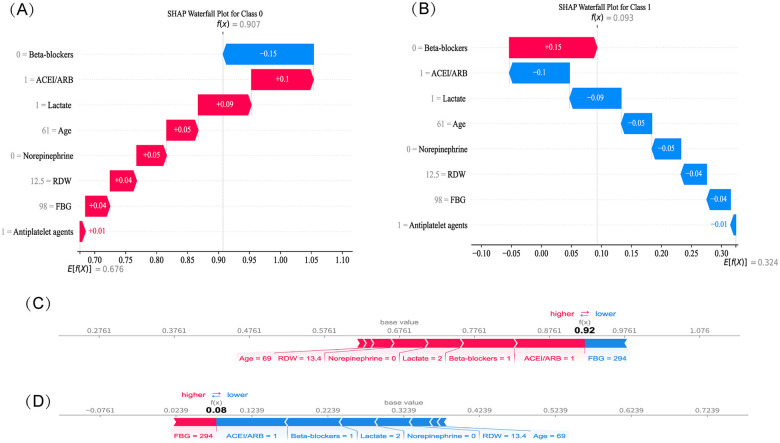
SHAP analysis results. **(A, B)** SHAP waterfall plots were used to explain personalized feature attributes for one patient. **(C, D)** SHAP force plots were used to explain personalized feature attributes for another patient.

Individual SHAP waterfall plots and force plots (Figure 5) were utilised to illustrate the granular feature contributions for specific patient predictions. For a representative patient predicted with a low risk of 28-day mortality (Figure 5A: predicted probability for Class 0 = 0.907 from a baseline E[f(X)] = 0.676), factors such as the use of ACEI/ARB (value = 1) and antiplatelet agents (value = 1), along with a normal FBG (98 mg/dL) and younger age (61 years), primarily contributed to this low-risk prediction. Conversely, the absence of Beta-blockers (value = 0) slightly increased the predicted risk. Another representative case (Figure 5D: predicted probability for Class 1 = 0.08 from a baseline E[f(X)] = 0.324) demonstrated a higher predicted risk of 28-day mortality, predominantly driven by a significantly elevated FBG (294 mg/dL). While the use of Beta-blockers (value = 1) and ACEI/ARB (value = 1) exerted associations with lower model-predicted mortality, their influence was outweighed by the substantial adverse impact of high FBG.

## Discussion

Our model should be understood as a bedside prognostic snapshot of AMI-MVA patients already presenting to the ICU, rather than an early-prediction tool for who will develop MVA. Several of the retained predictors — lactate, norepinephrine exposure, and 24-h treatment variables — are severity-reflective and represent downstream consequences of the same acute pathophysiological process being predicted. This has two consequences: first, the model's discriminative performance partly reflects concurrent severity rather than antecedent risk; second, the appropriate clinical use is triage of the already-admitted patient, not upstream population risk-stratification.

The present study developed and externally validated a machine learning-based model for predicting 28-day mortality in patients with AMI-MVA, addressing the lack of a comprehensive predictive framework in this high-risk population. Prior efforts—including nomogram-based risk identification (25, 26), random-forest frameworks with SHAP transparency (27), and nomograms incorporating electrical storm (19)—advanced the field incrementally but were each constrained by single-centre design, small samples, or absence of external validation. The SVM model developed herein achieved AUC 0.865 and 0.898 in internal and external validation, respectively, offering the most extensively validated framework yet reported for AMI-MVA mortality prediction.

SHAP analysis identified lactate, beta-blockers, and ACEI/ARB as the three most influential predictors, with FBG, RDW, norepinephrine, age, and antiplatelet agents contributing additional independent signal. These findings delineate what we term “metabolic crisis convergence”—a predictive construct in which markers of circulatory hypoperfusion (lactate), acute glucose dysregulation (FBG), and chronic hematopoietic stress (RDW) co-occur in patients at highest mortality risk. This construct should be interpreted as a prognostic signature reflecting multidimensional disease severity rather than a proven causal mechanism. Regarding lactate, Jentzer et al. (28) demonstrated that hypoperfusion markers more reliably stratify shock-related mortality than hemodynamic thresholds alone. Regarding FBG, Capes et al. (29) established that admission hyperglycemia independently predicts elevated 28-day mortality following AMI even in non-diabetic patients. Regarding RDW, Raval et al. (30) showed that elevated RDW in STEMI correlates with higher GRACE scores and 28-day mortality. By incorporating these three routinely accessible biomarkers, the present study addresses a gap in models that have historically overemphasised conventional cardiovascular risk factors while neglecting the metabolic crisis dimension central to AMI-MVA pathophysiology.

The negative SHAP contributions of beta-blockers and ACEI/ARB must be interpreted with caution. Registry data confirm beta-blocker benefit in ACS (31), but lower utilization in non-survivors almost certainly reflects confounding by indication: hemodynamically unstable patients cannot tolerate these agents, making reduced prescription a marker of severity rather than a causal driver of mortality. Norepinephrine similarly functions as a severity surrogate. At the predictive level, these variables retain robust marker value by reflecting hemodynamic stability and therapeutic tolerance.

The main model achieved AUC 0.865 (95% CI 0.821–0.907), and a sensitivity model excluding all treatment variables achieved AUC 0.801 (95% CI 0.748–0.851) — a difference of approximately 0.064 (Supplementary Figure S2). This gap represents the incremental discrimination attributable to treatment-exposure variables and, in a retrospective study, plausibly reflects prescribing patterns that co-vary with unmeasured severity rather than a causal treatment benefit. Patients receiving beta-blockers or ACEI/ARB during the first 24 h were, on average, hemodynamically stable enough to tolerate these agents, whereas patients in cardiogenic shock or on high-dose vasopressors typically did not receive them. Propensity-score matching and inverse-probability-of-treatment-weighting sensitivity analyses (Supplementary Section S10) attenuated the observed treatment-outcome associations but did not eliminate them, consistent with a mixture of genuine prognostic signal and residual confounding by indication. Both results — the ∼0.06 AUC gap and the adjusted associations — are shown transparently, and the model's contribution should be interpreted as risk stratification rather than causal treatment guidance.

Cross-continental external validation—training on a US MIMIC-IV cohort and testing on an independent Chinese cohort—is a methodological strength, as insufficient external validation has been identified as the dominant deficiency limiting clinical applicability of cardiovascular prediction models (32, 33). The external AUC of 0.898 and Brier score of 0.128 confirm that the model captures generalizable signals transcending system-specific treatment patterns.

The external cohort exhibited 28-day mortality of 53% vs. 32.5% in MIMIC-IV, likely reflecting case-selection (over-representation of patients transferred after failed initial management) and care-pathway differences (limited 24/7 primary PCI at the external site). Acceptable calibration across this mortality gradient supports transportability.

Compared with GRACE and TIMI, which demonstrate AUC values of approximately 0.72–0.74 in the AMI-MVA subgroup, the present model achieves approximately 15%–18% improvement—attributable to the structural absence of arrhythmia-specific variables, metabolic derangement markers, and critical care treatment-state information in conventional scoring systems. Chang et al. (34) and Hu et al. (35) confirmed the feasibility of embedding EHR-driven ML models within ICU clinical workflows for real-time critical cardiovascular risk prediction, supporting a stepwise translation pathway for the present model: initial deployment as a web-based risk calculator, subsequent EHR integration as a clinical decision support system, and ultimate prospective multicentre evaluation of whether model-guided stratification improves guideline-directed medication adherence and reduces AMI-MVA 28-day mortality.

The 15.4% PCI rate reflects both the intrinsic instability of AMI-MVA patients (in whom emergent catheterization is frequently deferred) and MIMIC-IV coding coverage (capturing only index-ICU-stay procedures). Our findings generalise primarily to patients too unstable for immediate reperfusion; extrapolation to those receiving timely primary PCI requires a separate PCI-treated validation cohort.

Limitations. (i) Retrospective design and modest external cohort. Both cohorts are retrospective and subject to unmeasured confounding, information bias in charting, and residual selection bias. The external validation set (*n* = 100) is modest; 95% CIs around AUC (0.835–0.949) and Brier score are correspondingly wide, and external-cohort subgroup analyses were not attempted. (ii) Prognostic snapshot, not early prediction. Several retained features (lactate, norepinephrine, treatment exposures) are downstream of the acute event; the model is a prognostic snapshot of already-critically-ill patients rather than an antecedent risk tool. (iii) Confounding by indication. Treatment-exposure variables likely reflect prescribing patterns that co-vary with unmeasured severity. PSM and IPTW sensitivity analyses attenuated but did not eliminate treatment-outcome associations. The treatment-free comparator model (AUC 0.801, 95% CI 0.748–0.851 internal; 0.771, 95% CI 0.675–0.853 external) is presented alongside the main model. (iv) Missing AMI-specific variables. Left ventricular ejection fraction was available for only 179/952 (18.8%) MIMIC-IV patients (median LVEF 50%, IQR 35%–55%); coronary anatomy, reperfusion modality, and door-to-balloon time were not available in harmonized form across cohorts. (v) Severity-score gap. Standardized scores (SOFA, APACHE II, SAPS II) are not routinely captured at the external site, precluding rigorous statistical adjustment for cross-site baseline severity differences. (vi) ICD-code validity. No chart-level audit of ICD-coded MVA events was performed; administrative coding of ventricular arrhythmia is known to encompass hemodynamically significant events and brief non-sustained VT alike. (vii) Imputation strategy. The main analysis imputed before the train/test split; training-only MICE sensitivity analysis (Supplementary Section S13) shows the effect is negligible (AUC difference <0.001). Lactate had a relatively high missingness rate (44.4%), which may reflect informative missingness (clinicians may be less likely to measure lactate in less acutely ill patients), violating the MICE missing-at-random assumption. The complete-case subset (*n* = 532) showed higher mortality (38.5%) than the full cohort (32.5%), consistent with preferential measurement in sicker patients. A complete-case sensitivity analysis (AUC 0.879 vs. 0.851; difference 0.028) mitigates but does not eliminate this concern. (viii) Additional limitations include time-invariant treatment coding (any 24-h exposure, not timing or dose), potential selection bias toward patients too unstable for immediate reperfusion, and restriction to two single-centre cohorts on two continents.

## Conclusion

The present study developed and externally validated an SVM-based machine learning model that predicts 28-day mortality in AMI-MVA patients across geographically and ethnically distinct populations. Integrating routinely available metabolic, treatment, and demographic predictors within a parsimonious eight-variable framework, supported by three-tier SHAP interpretability and comprehensive cross-continental validation, this model provides a robust and transparent candidate tool for early bedside risk stratification within the first 24 h of ICU admission in this high-mortality population.

## Data Availability

The data analyzed in this study is subject to the following licenses/restrictions: The MIMIC-IV database is publicly available but requires registration and approval from PhysioNet. Access is restricted to registered users who have completed the required data use agreement and training certification. The Maoming People's Hospital dataset is restricted to approved researchers and cannot be shared publicly due to patient privacy protection regulations and institutional data governance policies. Both datasets have been de-identified and anonymized prior to analysis. The de-identified data from this study are available upon reasonable request from the corresponding author, subject to ethics committee approval and institutional data sharing agreements. Requests to access these datasets should be directed to Requests for access to the de-identified dataset from this study can be directed to the corresponding author, Jiechun Yao (673589060@qq.com). Data access requests will be reviewed and approved by the Ethics Committee of Maoming People's Hospital in accordance with institutional data sharing policies and the original ethics approval conditions.
