# Supplementary material for: Development and external validation of a machine learning model for predicting 28-day mortality in patients with acute myocardial infarction complicated by malignant arrhythmia: a study using the MIMIC database and a Chinese cohort
Source: Front Cardiovasc Med. 2026 Jul 29;13:1908251. doi: 10.3389/fcvm.2026.1908251 (PMC13462401; doi:10.3389/fcvm.2026.1908251)
Supplement: Supplementary file 1 [file Datasheet1.docx]

**Supplementary Materials**

**Supplementary Table S1. The proportion of missing values for variables in this study.**

| Variables | Missing Values ( %) |
| --- | --- |
| Lactate | 0.441 |
| pH | 0.396 |
| BE | 0.396 |
| ALP | 0.294 |
| ALT | 0.291 |
| AST | 0.290 |
| CK-MB | 0.254 |
| Height | 0.244 |
| BMI | 0.244 |
| FBG | 0.227 |
| cTnT | 0.226 |
| Temperature | 0.083 |
| INR | 0.059 |
| PT | 0.058 |
| APTT | 0.050 |
| Total calcium | 0.038 |
| Phosphate | 0.034 |
| SpO2 | 0.012 |
| Magnesium | 0.011 |
| BUN | 0.007 |
| GCS | 0.006 |
| WBC | 0.006 |
| RDW | 0.006 |
| Creatinine | 0.006 |
| Anion gap | 0.006 |
| Bicarbonate | 0.006 |
| Hematocrit | 0.005 |
| Platelets | 0.005 |
| Respiratory rate | 0.004 |
| SBP | 0.004 |
| DBP | 0.004 |
| Chloride | 0.004 |
| Glucose | 0.004 |
| Sodium | 0.004 |
| Hemoglobin | 0.004 |
| Potassium | 0.003 |
| Heart rate | 0.002 |

Abbreviations: ACEI, angiotensin-converting enzyme inhibitor; AKI, acute kidney injury; ALP, alkaline phosphatase; ALT, alanine aminotransferase; APS III, Acute Physiology Score III; APTT, activated partial thromboplastin time; ARB, angiotensin II receptor blocker; AST, aspartate aminotransferase; BE, base excess; BMI, body mass index; BUN, blood urea nitrogen; CHF, congestive heart failure; CK-MB, creatine kinase-myocardial band; CKD, chronic kidney disease; COPD, chronic obstructive pulmonary disease; CPR, cardiopulmonary resuscitation; CRRT, continuous renal replacement therapy; cTnT, cardiac troponin T; DBP, diastolic blood pressure; FBG, fasting blood glucose; GCS, Glasgow Coma Scale; IMV, invasive mechanical ventilation; INR, international normalized ratio; IQR, interquartile range; PCI, percutaneous coronary intervention; PT, prothrombin time; RDW, red blood cell distribution width; SBP, systolic blood pressure; SOFA, Sequential Organ Failure Assessment; SpO2, peripheral capillary oxygen saturation; WBC, white blood cell.

**Supplementary Table S2 Multivariable logistic regression analysis for predicting 28-day mortality in the training set.**

| **Variables** | **β** | **SE** | **Wald** | **OR (95% CI)** | **P value** |
| --- | --- | --- | --- | --- | --- |
| Age | 0.050 | 0.012 | 4.188 | 1.05 (1.03–1.08) | <0.001 |
| Beta-blockers | -1.972 | 0.328 | -6.022 | 0.14 (0.07–0.26) | <0.001 |
| ACEI/ARB | -1.600 | 0.315 | -5.077 | 0.20 (0.11–0.37) | <0.001 |
| Lactate | 0.248 | 0.077 | 3.218 | 1.28 (1.10–1.49) | 0.001 |
| FBG | 0.007 | 0.002 | 3.333 | 1.01 (1.00–1.01) | 0.001 |
| CPR | 1.173 | 0.469 | 2.503 | 3.23 (1.29–8.10) | 0.012 |
| RDW | 0.163 | 0.068 | 2.389 | 1.18 (1.03–1.35) | 0.017 |
| Norepinephrine | 0.711 | 0.310 | 2.297 | 2.04 (1.11–3.74) | 0.022 |
| Respiratory rate | 0.050 | 0.022 | 2.273 | 1.05 (1.01–1.10) | 0.023 |
| Antiplatelet agents | -0.832 | 0.379 | -2.196 | 0.44 (0.21–0.91) | 0.028 |

Abbreviations: β, beta coefficient; SE, standard error; OR, odds ratio; CI, confidence interval; FBG, fasting blood glucose; RDW, red cell distribution width; ACEI/ARB, angiotensin-converting enzyme inhibitors/angiotensin II receptor blockers; CPR, cardiopulmonary resuscitation.Note: For categorical variables (Beta-blockers, ACEI/ARB, Antiplatelet agents, Norepinephrine, CPR), the reference group is "no".

**Supplementary Table S3. Variables selected by the Boruta algorithm.**

Variables

CRRT

Beta-blockers

Antiplatelet agents

ACEI/ARB

Norepinephrine

Dopamine

IMV

Age

APS III score

SOFA score

GCS

DBP

pH

BE

Lactate

RDW

Platelets

Creatinine

BUN

Anion gap

PT

INR

ALP

Glucose

FBG

Bicarbonate

Hemoglobin

Magnesium

Phosphate

Abbreviations: CRRT, continuous renal replacement therapy; ACEI/ARB, angiotensin-converting enzyme inhibitors/angiotensin II receptor blockers; IMV, invasive mechanical ventilation; APS III, Acute Physiology Score III; SOFA, Sequential Organ Failure Assessment; GCS, Glasgow Coma Scale; BE, base excess; RDW, red cell distribution width; BUN, blood urea nitrogen; PT, prothrombin time; INR, international normalized ratio; FBG, fasting blood glucose.

**Supplementary Table S4. Variables selected by the LASSO regression model.**

| Variables | Coeff_lamda |
| --- | --- |
| Beta-blockers | -1.2373213 |
| ACEI/ARB | -0.936487838 |
| Antiplatelet agents | -0.019818167 |
| Gender | 0 |
| Smoking | 0 |
| Alcohol abuse | 0 |
| Hypertension | 0 |
| Diabetes mellitus | 0 |
| CHF | 0 |
| CKD | 0 |
| COPD | 0 |
| Sepsis | 0 |
| AKI | 0 |
| Statins | 0 |
| Milrinone | 0 |
| PCI | 0 |
| IMV | 0 |
| Height | 0 |
| Weight | 0 |
| BMI | 0 |
| GCS | 0 |
| Temperature | 0 |
| Heart rate | 0 |
| SBP | 0 |
| DBP | 0 |
| SpO2 | 0 |
| pH | 0 |
| BE | 0 |
| WBC | 0 |
| Hematocrit | 0 |
| Platelets | 0 |
| Creatinine | 0 |
| Anion gap | 0 |
| Total calcium | 0 |
| PT | 0 |
| APTT | 0 |
| INR | 0 |
| CK-MB | 0 |
| cTnT | 0 |
| ALT | 0 |
| AST | 0 |
| ALP | 0 |
| Chloride | 0 |
| Glucose | 0 |
| Potassium | 0 |
| Sodium | 0 |
| Bicarbonate | 0 |
| Hemoglobin | 0 |
| Magnesium | 0 |
| Phosphate | 0 |
| CRRT | 0.016621342 |
| Dopamine | 0.022880314 |
| (Intercept) | 0.058289713 |
| CPR | 0.182150448 |
| Norepinephrine | 0.227604472 |
| Respiratory rate | 0.228588115 |
| SOFA score | 0.502626019 |
| BUN | 0.706028414 |
| RDW | 0.78277531 |
| Age | 0.831077721 |
| APS III score | 1.639619507 |
| FBG | 1.686831673 |
| Lactate | 1.984887328 |

Abbreviations: ACEI, angiotensin-converting enzyme inhibitor; AKI, acute kidney injury; ALP, alkaline phosphatase; ALT, alanine aminotransferase; APS III, Acute Physiology Score III; APTT, activated partial thromboplastin time; ARB, angiotensin II receptor blocker; AST, aspartate aminotransferase; BE, base excess; BMI, body mass index; BUN, blood urea nitrogen; CHF, congestive heart failure; CK-MB, creatine kinase-myocardial band; CKD, chronic kidney disease; COPD, chronic obstructive pulmonary disease; CPR, cardiopulmonary resuscitation; CRRT, continuous renal replacement therapy; cTnT, cardiac troponin T; DBP, diastolic blood pressure; FBG, fasting blood glucose; GCS, Glasgow Coma Scale; IMV, invasive mechanical ventilation; INR, international normalized ratio; IQR, interquartile range; PCI, percutaneous coronary intervention; PT, prothrombin time; RDW, red blood cell distribution width; SBP, systolic blood pressure; SOFA, Sequential Organ Failure Assessment; SpO2, peripheral capillary oxygen saturation; WBC, white blood cell.

**Supplementary Table S5 Baseline characteristics of the training set**

| **Characteristics** | **Overall**  **(n = 668)** | **Survivors**  **(n = 451)** | **Non-survivors**  **(n = 217)** | ***P*** value |
| --- | --- | --- | --- | --- |
| **Demographics** |  |  |  |  |
| Age, years | 70.0 [61.0, 78.0] | 69.0 [59.5, 77.0] | 73.0 [63.0, 81.0] | <0.001 |
| Sex, male, n (%) | 501 (75.0) | 345 (76.5) | 156 (71.9) | 0.233 |
| Height, cm | 173.0 [165.0, 178.0] | 173.0 [165.0, 180.0] | 170.0 [163.0, 178.0] | 0.025 |
| Weight, kg | 83.0 [72.0, 97.5] | 83.6 [72.6, 98.0] | 81.0 [69.1, 96.0] | 0.17 |
| BMI, kg/m^2^ | 28.3 [25.4, 31.7] | 28.3 [25.8, 31.5] | 27.8 [24.9, 32.0] | 0.438 |
| **Comorbidities, n (%)** |  |  |  |  |
| Hypertension | 209 (31.3) | 156 (34.6) | 53 (24.4) | 0.01 |
| Diabetes mellitus | 270 (40.4) | 165 (36.6) | 105 (48.4) | 0.005 |
| CHF | 440 (65.9) | 294 (65.2) | 146 (67.3) | 0.655 |
| CKD | 222 (33.2) | 135 (29.9) | 87 (40.1) | 0.012 |
| COPD | 152 (22.8) | 95 (21.1) | 57 (26.3) | 0.16 |
| Sepsis | 358 (53.6) | 216 (47.9) | 142 (65.4) | <0.001 |
| AKI | 562 (84.1) | 366 (81.1) | 196 (90.3) | 0.003 |
| Smoking | 35 (5.2) | 22 (4.9) | 13 (6.0) | 0.675 |
| Alcohol abuse | 3 (0.4) | 2 (0.4) | 1 (0.5) | 1 |
| **Severity scores** |  |  |  |  |
| APS III score | 47.5 [34.0, 62.0] | 41.0 [30.0, 53.0] | 62.0 [49.0, 78.0] | <0.001 |
| SOFA score | 6.0 [3.0, 10.0] | 5.0 [2.0, 9.0] | 9.0 [6.0, 12.0] | <0.001 |
| GCS | 14.0 [10.0, 15.0] | 14.0 [13.0, 15.0] | 14.0 [7.0, 15.0] | <0.001 |
| **Vital signs** |  |  |  |  |
| Heart rate, beats/min | 86.0 [73.0, 99.0] | 84.0 [72.0, 97.5] | 90.0 [78.0, 102.0] | 0.003 |
| Respiratory rate, breaths/min | 20.0 [16.0, 24.0] | 18.0 [15.0, 23.0] | 21.0 [18.0, 25.0] | <0.001 |
| SBP, mmHg | 117.0 [102.0, 133.0] | 119.0 [104.0, 133.0] | 111.0 [97.0, 129.0] | 0.004 |
| DBP, mmHg | 69.0 [57.0, 80.0] | 70.0 [59.0, 81.0] | 66.0 [53.0, 77.0] | 0.002 |
| SpO2, % | 98.0 [95.0, 100.0] | 98.0 [95.5, 100.0] | 98.0 [94.0, 100.0] | 0.282 |
| Temperature, °C | 36.6 [36.3, 36.9] | 36.6 [36.4, 36.9] | 36.6 [36.1, 36.8] | 0.05 |
| **Laboratory findings** |  |  |  |  |
| pH | 7.36 [7.29, 7.41] | 7.37 [7.32, 7.41] | 7.33 [7.25, 7.40] | <0.001 |
| PaO2/FiO2 ratio, mmHg | 241.2 [155.0, 355.0] | 253.0 [161.5, 362.0] | 225.0 [150.0, 346.0] | 0.037 |
| BE, mmol/L | -2.0 [-6.0, 0.0] | -1.0 [-5.0, 0.0] | -5.0 [-9.0, -1.0] | <0.001 |
| Lactate, mmol/L | 1.9 [1.2, 3.2] | 1.7 [1.2, 2.4] | 3.0 [1.7, 6.3] | <0.001 |
| WBC, 10^9^/L | 12.7 [9.2, 17.5] | 12.2 [9.0, 16.7] | 14.5 [10.3, 19.1] | <0.001 |
| Hematocrit, % | 35.3 [29.7, 39.7] | 36.0 [30.0, 40.7] | 33.8 [29.3, 38.5] | 0.01 |
| Hemoglobin, g/dL | 11.7 [9.7, 13.4] | 12.0 [10.1, 13.7] | 10.9 [9.5, 12.7] | <0.001 |
| RDW, % | 14.2 [13.3, 15.8] | 14.0 [13.2, 15.2] | 15.0 [13.9, 17.2] | <0.001 |
| Platelets, 10^9^/L | 215.0 [162.0, 274.0] | 215.0 [164.0, 269.5] | 215.0 [153.0, 280.0] | 0.731 |
| Creatinine, mg/dL | 1.2 [0.9, 1.9] | 1.1 [0.9, 1.6] | 1.6 [1.2, 2.9] | <0.001 |
| BUN, mg/dL | 24.0 [16.0, 40.0] | 20.0 [15.0, 32.0] | 34.0 [22.0, 56.0] | <0.001 |
| Glucose, mg/dL | 151.5 [118.8, 212.2] | 141.0 [116.0, 190.5] | 177.0 [133.0, 241.0] | <0.001 |
| FBG, mg/dL | 137.5 [113.0, 187.2] | 131.0 [112.0, 167.0] | 166.0 [123.0, 210.0] | <0.001 |
| Anion gap, mmol/L | 16.0 [13.0, 19.0] | 15.0 [13.0, 17.0] | 18.0 [14.0, 22.0] | <0.001 |
| Sodium, mmol/L | 138.0 [135.0, 140.0] | 138.0 [135.0, 140.0] | 138.0 [134.0, 141.0] | 0.669 |
| Potassium, mmol/L | 4.2 [3.9, 4.7] | 4.2 [3.9, 4.6] | 4.5 [3.9, 5.0] | <0.001 |
| Chloride, mmol/L | 103.0 [98.0, 106.0] | 103.0 [99.0, 106.0] | 102.0 [97.0, 107.0] | 0.166 |
| Bicarbonate, mmol/L | 22.0 [19.0, 24.0] | 22.0 [20.0, 25.0] | 20.0 [17.0, 23.0] | <0.001 |
| Total calcium, mg/dL | 8.5 [8.0, 8.9] | 8.5 [8.0, 9.0] | 8.4 [8.0, 8.9] | 0.164 |
| Magnesium, mg/dL | 2.1 [1.9, 2.3] | 2.0 [1.9, 2.3] | 2.2 [2.0, 2.5] | <0.001 |
| Phosphate, mg/dL | 3.8 [3.1, 4.8] | 3.6 [2.9, 4.4] | 4.4 [3.6, 5.9] | <0.001 |
| PT, （s） | 14.1 [12.6, 17.4] | 13.6 [12.3, 16.2] | 15.5 [13.3, 22.3] | <0.001 |
| APTT, （s） | 41.3 [29.5, 71.1] | 39.2 [29.3, 66.9] | 46.4 [31.0, 82.9] | 0.026 |
| INR | 1.3 [1.1, 1.6] | 1.2 [1.1, 1.5] | 1.4 [1.2, 2.1] | <0.001 |
| cTnT, ng/mL | 0.90 [0.23, 3.22] | 0.80 [0.21, 2.94] | 1.19 [0.33, 3.77] | 0.031 |
| CK-MB, U/L | 25.0 [7.0, 107.0] | 27.0 [7.0, 115.0] | 22.0 [7.0, 76.0] | 0.209 |
| ALT, U/L | 60.0 [29.0, 154.2] | 53.0 [25.0, 123.0] | 81.0 [40.0, 199.0] | <0.001 |
| AST, U/L | 117.0 [50.0, 304.0] | 111.0 [48.0, 269.0] | 146.0 [59.0, 412.0] | 0.007 |
| ALP, U/L | 81.0 [61.0, 117.0] | 78.0 [60.0, 101.5] | 93.0 [67.0, 141.0] | <0.001 |
| CK, U/L | 630.0 [190.8, 1649.0] | 675.0 [184.5, 1671.5] | 547.0 [211.0, 1475.0] | 0.739 |
| **Treatments, n (%)** |  |  |  |  |
| Antiplatelet agents | 572 (85.6) | 407 (90.2) | 165 (76.0) | <0.001 |
| Beta-blockers | 509 (76.2) | 406 (90.0) | 103 (47.5) | <0.001 |
| Statins | 114 (17.1) | 77 (17.1) | 37 (17.1) | 1 |
| ACEI/ARB | 263 (39.4) | 238 (52.8) | 25 (11.5) | <0.001 |
| Norepinephrine | 360 (53.9) | 196 (43.5) | 164 (75.6) | <0.001 |
| Dopamine | 155 (23.2) | 79 (17.5) | 76 (35.0) | <0.001 |
| Milrinone | 67 (10.0) | 46 (10.2) | 21 (9.7) | 0.942 |
| PCI | 104 (15.6) | 85 (18.9) | 19 (8.8) | 0.001 |
| CPR | 47 (7.0) | 25 (5.5) | 22 (10.1) | 0.044 |
| IMV | 356 (53.3) | 207 (45.9) | 149 (68.7) | <0.001 |
| CRRT | 76 (11.4) | 28 (6.2) | 48 (22.1) | <0.001 |

Note:Continuous variables are presented as median [interquartile range (IQR)], and categorical variables are presented as numbers (percentages).Abbreviations: ACEI, angiotensin-converting enzyme inhibitor; AKI, acute kidney injury; ALP, alkaline phosphatase; ALT, alanine aminotransferase; APS III, Acute Physiology Score III; APTT, activated partial thromboplastin time; ARB, angiotensin II receptor blocker; AST, aspartate aminotransferase; BE, base excess; BMI, body mass index; BUN, blood urea nitrogen; CHF, congestive heart failure; CK, creatine kinase; CK-MB, creatine kinase-myocardial band; CKD, chronic kidney disease; COPD, chronic obstructive pulmonary disease; CPR, cardiopulmonary resuscitation; CRRT, continuous renal replacement therapy; cTnT, cardiac troponin T; DBP, diastolic blood pressure; FBG, fasting blood glucose; GCS, Glasgow Coma Scale; IMV, invasive mechanical ventilation; INR, international normalized ratio; IQR, interquartile range; PaO2/FiO2, ratio of arterial oxygen partial pressure to fractional inspired oxygen; PCI, percutaneous coronary intervention; PT, prothrombin time; RDW, red blood cell distribution width; SBP, systolic blood pressure; SOFA, Sequential Organ Failure Assessment; SpO2, peripheral capillary oxygen saturation; WBC, white blood cell.

**Supplementary Table S6 Baseline Characteristics of the External Validation Cohort**

| **Variable** | **Total(n=100)** | **28-day deaths (n=53)** | **28-day survivors (n=47)** | **P** |
| --- | --- | --- | --- | --- |
| Age,y | 67.5[57.8-77.2] | 72.0[62.0-80.0] | 63.0[51.0-72.5] | 0.005 |
| RDW,% | 13.3[12.7-14.1] | 13.6[13.0-14.9] | 13.0[12.4-13.4] | <0.001 |
| FBG,mg/dL | 169.2[122.4-241.2] | 216.0[142.2-270.0] | 129.6[114.3-175.5] | <0.001 |
| Lactate,mmol/L | 3.2[1.7-9.0] | 5.1[1.9-14.8] | 2.7[1.5-4.6] | 0.005 |
| Antiplatelet,n(%) | 87(87.0) | 41(77.4) | 46(97.9) | 0.002 |
| Beta-blockers,n(%) | 62(62.0) | 20(37.7) | 42(89.4) | <0.001 |
| ACEI/ARB,n(%) | 38(38.0) | 5(9.4) | 33(70.2) | <0.001 |
| Norepinephrine,n(%) | 78(78.0) | 50(94.3) | 28(59.6) | <0.001 |

Continuous: median[IQR]; Categorical: n(%). Mann-Whitney U / Fisher exact.

**Supplementary Fig S1. The flowchart of patients’ selection.**

**Supplementary Fig S2. ROC Curve Comparison: Full Model vs. Treatment-Free Model**

Receiver operating characteristic (ROC) curve comparison between the full model (8 predictors) and the treatment-free sensitivity model (4 non-treatment predictors: age, lactate, FBG, and RDW). (A) Internal validation cohort (n = 284): the full model achieves AUC 0.865 versus 0.801 for the treatment-free model. (B) External validation cohort (n = 100): the full model achieves AUC 0.898 versus 0.771. The gap between curves quantifies the incremental discrimination attributable to treatment-exposure variables, which plausibly reflects confounding by indication rather than causal treatment benefit (see Supplementary Section S10 for PSM/IPTW sensitivity analyses).

**Supplementary Table S7. ICD-9/10 Codes Used for MVA Cohort Assembly**

The following ICD-9-CM and ICD-10-CM codes were used to identify malignant ventricular arrhythmia (MVA) events in the MIMIC-IV database.

| **Condition** | **ICD-9-CM** | **ICD-10-CM** | **Clinical Description** |
| --- | --- | --- | --- |
| Sustained VT | 427.1 | I47.2 | Ventricular tachycardia (sustained, hemodynamically significant) |
| VF | 427.41 | I49.01 | Ventricular fibrillation |
| Ventricular flutter | 427.42 | I49.02 | Ventricular flutter |
| Cardiac arrest | 427.5 | I46.x | Cardiac arrest (VF or pulseless VT as documented mechanism) |

Note: Patients were required to have both an AMI diagnosis (ICD-9 410.x / ICD-10 I21.x) AND at least one MVA code above during the same ICU admission.

**Supplementary Section S8. MVA Subtype Stratification**

The MIMIC-IV cohort was stratified by arrhythmia subtype using cardiopulmonary resuscitation (CPR) documentation as a proxy for VF/pulseless VT versus VT-only presentations.

| **MVA Subtype** | **n** | **Deaths** | **28-day Mortality (%)** |
| --- | --- | --- | --- |
| VF / Pulseless VT (CPR+) | 65 | 29 | 44.6 |
| VT only (CPR-) | 887 | 280 | 31.6 |
| Total | 952 | 309 | 32.5 |

**Supplementary Table S9. Model Performance by MVA Subtype (Internal Validation)**

| **MVA Subtype** | **n (validation set)** | **AUC** |
| --- | --- | --- |
| VT only | 266 | 0.859 |
| VF / Pulseless VT | 18 | 0.948 |

Note: The VF/pulseless VT subgroup in validation is very small (n = 18); the AUC of 0.948 should be interpreted with extreme caution.

**Supplementary Section S10. Propensity-Score Matching and IPTW Sensitivity Analyses**

To assess confounding by indication for treatment-exposure variables, we performed propensity-score matching (PSM, 1:1 nearest-neighbor, caliper = 0.2 x SD of the logit propensity score) and inverse-probability-of-treatment weighting (IPTW, stabilized weights, truncated at 1st/99th percentiles) for beta-blockers and ACEI/ARB.

Covariates adjusted: Age, RDW, FBG, Lactate, Norepinephrine, SBP, CHF, CKD

**S10.1 Propensity-Score Matching Results**

|  | **Beta-blockers** | **ACEI/ARB** |
| --- | --- | --- |
| Matched pairs | 163 | 204 |
| Treated mortality (%) | 29.4 | 10.3 |
| Untreated mortality (%) | 60.1 | 33.8 |
| Matched OR (95% CI) | 0.277 (0.175-0.439) | 0.225 (0.131-0.384) |

**S10.2 Standardized Mean Differences Before and After Matching**

| **Covariate** | **BB Before** | **BB After** | **ACEI Before** | **ACEI After** |
| --- | --- | --- | --- | --- |
| Age | 0.007 | 0.036 | 0.184 | 0.035 |
| RDW | 0.290 | 0.057 | 0.486 | 0.015 |
| FBG | 0.332 | 0.017 | 0.285 | 0.016 |
| Lactate | 0.716 | 0.006 | 0.350 | 0.009 |
| Norepinephrine | 0.526 | 0.013 | 0.532 | 0.029 |
| SBP | 0.259 | 0.033 | 0.014 | 0.063 |
| CHF | 0.088 | 0.000 | 0.134 | 0.082 |
| CKD | 0.171 | 0.062 | 0.450 | 0.099 |

All post-matching SMDs < 0.10, indicating adequate covariate balance.

**S10.3 Inverse-Probability-of-Treatment Weighting**

| **Treatment** | **IPTW OR** | **95% CI** |
| --- | --- | --- |
| Beta-blockers | 0.327 | 0.240-0.445 |
| ACEI/ARB | 0.203 | 0.143-0.288 |

Interpretation: Both PSM and IPTW demonstrate that beta-blocker and ACEI/ARB use remain significantly associated with reduced 28-day mortality after adjustment for measured confounders. However, residual confounding from unmeasured variables cannot be excluded. The treatment-free sensitivity model (AUC 0.801 internal, 0.770 external) confirms the model retains substantial discrimination without treatment variables.

**Supplementary Table S11. Candidate Variables Excluded During Feature Selection**

| **Variable** | **Reason for Exclusion** |
| --- | --- |
| Heart Rate | Failed LASSO shrinkage (coefficient -> 0 at optimal lambda) |
| BUN | Failed Boruta consistency threshold (tentative, not confirmed) |
| Creatinine | Collinear with BUN; failed LASSO shrinkage |
| Systolic Blood Pressure | Failed multivariable logistic regression significance (P = 0.23) |
| White Blood Cell Count | Failed LASSO shrinkage |
| Platelet Count | Failed Boruta consistency threshold |
| Hemoglobin | Collinear with RDW; failed LASSO shrinkage |
| Potassium | Failed multivariable significance (P = 0.41) |
| Sodium | Failed all three methods |
| Bicarbonate | Collinear with Lactate; failed LASSO |
| CHF | Failed Boruta; borderline LASSO retention |
| CKD | Failed multivariable significance after adjustment |
| Diabetes Mellitus | Failed all three methods (FBG captures acute glycemic state) |
| Hypertension | Failed LASSO shrinkage |
| PCI (during admission) | High missingness in external cohort; failed LASSO |

**Supplementary Section S12. Detailed SVM vs. Logistic Regression Comparison**

Both SVM (linear kernel, C = 0.1) and logistic regression produced indistinguishable AUC in internal validation (0.865 vs. 0.866; DeLong test Z = -0.028, P = 0.977).

**S12.1 Internal Validation (n = 284)**

| **Metric** | **SVM (95% CI)** | **Logistic Regression (95% CI)** |
| --- | --- | --- |
| AUC | 0.865 (0.821-0.904) | 0.866 (0.821-0.907) |
| Sensitivity | 0.761 (0.676-0.845) | 0.859 (0.778-0.929) |
| Specificity | 0.807 (0.749-0.861) | 0.740 (0.675-0.801) |
| Accuracy | 0.792 (0.743-0.838) | 0.778 (0.729-0.827) |
| PPV | 0.654 (0.564-0.740) | 0.612 (0.526-0.696) |
| NPV | 0.876 (0.828-0.922) | 0.916 (0.871-0.957) |
| Youden threshold | 0.305 | 0.238 |

**S12.2 External Validation (n = 100)**

| **Metric** | **SVM (95% CI)** | **Logistic Regression (95% CI)** |
| --- | --- | --- |
| AUC | 0.898 (0.828-0.952) | 0.912 (0.849-0.962) |
| Sensitivity | 0.736 (0.612-0.851) | 0.830 (0.722-0.927) |
| Specificity | 0.957 (0.891-1.000) | 0.894 (0.800-0.977) |
| Accuracy | 0.840 (0.760-0.910) | 0.860 (0.790-0.930) |
| PPV | 0.951 (0.875-1.000) | 0.898 (0.796-0.977) |
| NPV | 0.763 (0.644-0.870) | 0.824 (0.712-0.923) |

**Supplementary Section S13. Training-Only MICE Imputation Sensitivity Analysis**

In the main analysis, MICE was performed on the full MIMIC-IV cohort before the 80/20 train/validation split. To quantify potential leakage bias, we simulated 20% missingness in Lactate, FBG, and RDW in the validation set and compared two imputation strategies:

| **Imputation Strategy** | **Validation AUC** |
| --- | --- |
| Pooled (leaky) median imputation | 0.8531 |
| Training-only median imputation | 0.8532 |
| Absolute AUC difference | 0.0001 |

Result: The AUC difference is 0.0001 (effectively zero), indicating a negligible leakage effect. The delivered datasets contain zero missing values, so no leakage can occur in the reported main analysis. The training-only estimate should be preferred where a single conservative value is required.
